# Case Report: First Evidence of a Benign Bone Cyst in an Adult Teckel Dog Treated With Shark Teeth-Derived Bioapatites

**DOI:** 10.3389/fvets.2021.626992

**Published:** 2021-02-22

**Authors:** Mario García-González, Fernando María Muñoz Guzón, Antonio González-Cantalapiedra, Mónica López-Peña, Felipe de Frutos Pachón, Teresa Pereira-Espinel Plata, Pío Manuel González Fernández, Julia Asunción Serra Rodríguez

**Affiliations:** ^1^Clinical Sciences Department, Veterinary Faculty, Universidade de Santiago de Compostela, Lugo, Spain; ^2^Clínica Veterinaria El Parque, Talavera de la Reina, Spain; ^3^New Materials Group, Department of Applied Physics, Galicia Sur Health Research Institute (IISGS), Universidade de Vigo, Vigo, Spain

**Keywords:** eco-friendly biomaterial, bone cyst, bone regeneration, marine scaffold, marine biomaterial, bioapatites, regenerative medicine, Teckel dog

## Abstract

Bone cysts are a very rare orthopedic pathology in veterinary medicine, the general prevalence of which is unknown. A unicameral bone cyst was diagnosed in an adult female Teckel dog with a limp that was treated surgically by filling the defect with marine bioapatites. The treatment was effective and at 8 weeks the defect had remodeled 50.24%. Eighteen months after surgery, the defect had remodeled 94.23%. The limp disappeared after surgery, and functional recovery was good in all stages after surgery. No adverse reactions were observed at the local or systemic level. This is the first report of a benign bone cyst in an lame adult female Teckel successfully treated with a novel marine bioapatite.

## Introduction

A bone cyst is a benign intraosseous tumor-like lesion covered by a thin layer of connective tissue and containing a clear to serosanguineous fluid (1). Bone cysts are classified as either aneurysmal or unicameral (simple). Simple bone cysts may be unicameral (UBC) or partially separated (2). The general prevalence of this tumor-like lesion in dogs is unknown because it is a poorly studied pathology. In humans, bone cysts represent ~3% of all bone tumors (1, 3, 4). They develop most frequently within the proximal or distal metaphysis of the long tubular bones, adjacent to the growth plate, migrating away as bone growth occurs (5).

Bone cysts are commonly diagnosed in young dogs (5–15 months) and are more frequently found in medium-sized and large dogs, suspecting an hereditary condition in Dobermann and Old English Sheepdog (1, 5, 6). Cases have been found in Mastiffs, Golden Retrievers (7), German Shepherds, Shih Tzus (8), Siberian Huskies (9), and Yorkshire Terriers (10). This pathology affects males more commonly than females (10).

The treatment requires surgical intervention and is usually treated with open curettage and bone graft. Sometimes an internal fixation dispositive is necessary. The choice of bone filling depends on several factors, these being the characteristics of the patient, location and size of the bone defect, and the risk of fracture. Types of bone fillers include allografts, autografts, bone cements, and commercial synthetic fillers. In this type of pathology, the bone filler most used is the allograft, because it offers few complications and an unlimited amount of graft and avoids the morbidity of the donor site (11). The use of autograft presents limitations as to the amount of bone and the morbidity of the donor site. Also, the use of this type of bone filler may lead to possible infections, scar formation, or hematoma (12).

Within synthetic bone fillers, recent studies have discovered the possibility of obtaining bone grafts of marine origin, e.g., BIOFAST-VET (BV). This novel product was designed to repair and regenerate bone tissue. It is made from a ceramic material obtained from food waste, the shark teeth (*Prionace glauca*). Its strengths are that it is a very abundant material today and the cost is low (13–15). This marine bioapatite has been successfully used in a preliminary study in dogs and cats for the treatment of fractures and arthrodesis carried out by the same research groups as this research (16).

The purpose of this study, compared with the preliminary study, is to present a full and comprehensive description, including the long-term follow-up, of the first preliminary report of a benign bone cyst in an adult female Teckel. The involvement in adult females of the Teckel breed is unknown, and the literature has not reported any cases yet. No cases of bone cyst have been reported in which marine bioapatites have been used for their treatment. Within this purpose, the objective of this study involves an exhaustive description of this first report and evaluate the effectivity of this marine bioapatite as a surgical treatment.

This material has shown promising results in previous *in vivo* and *in vitro* studies (13–15) and in a preliminary clinical trial in dogs and cats (16). *In vivo* results performed in rats revealed a significant increase in bone mineral density compared with a synthetic control. In *in vitro* studies, a greater osteogenic potential was found compared with other commercial grafts. The composition, morphology, and characterization (Raman and XRD techniques) of shark teeth–derived bioapatites have been studied and revealed a globular porous structure ~70% composed of biphasic apatites (HA, apatite-CaP, fluorapatite) and ~30% non-apatites phase (whitlockite, b-TCP) in addition to contributions of F, Na, and Mg (13–15).

The fabrication method of the bone substitute of marine origin, BV, is based on pyrolytic techniques in order to remove the organic compounds. The natural precursor (shark teeth *Prionace glauca*) is heated to 950°C for 12 h using a heating ramp of 2°C per min and a cooling ramp of 20°C/min, as described elsewhere (13, 14). Once pyrolyzed, the powders were subjected to a sieving process to select macrogranules in the diameter range of 0.5–1.0 mm, 1.0–2.0 mm, and 2.0–3.0 mm. The sterilization method used was Gamma radiation (Aragogamma S.L.) (13–15).

## Case Report

The guidelines of the CARE declaration have been followed in the preparation of this case report (see the checklist in Supplementary Table 2).

A 2-year-old sterilized female Teckel (8 kg) was presented with several days' lameness (Supplementary Video 1). Physical examination showed the presence of an inflamed area at the level of the right distal humeral region without excessive pain. The patient showed no further abnormalities. In the medical history, no other pathologies, genetic information, or interventions have been found.

**Figure 1:**
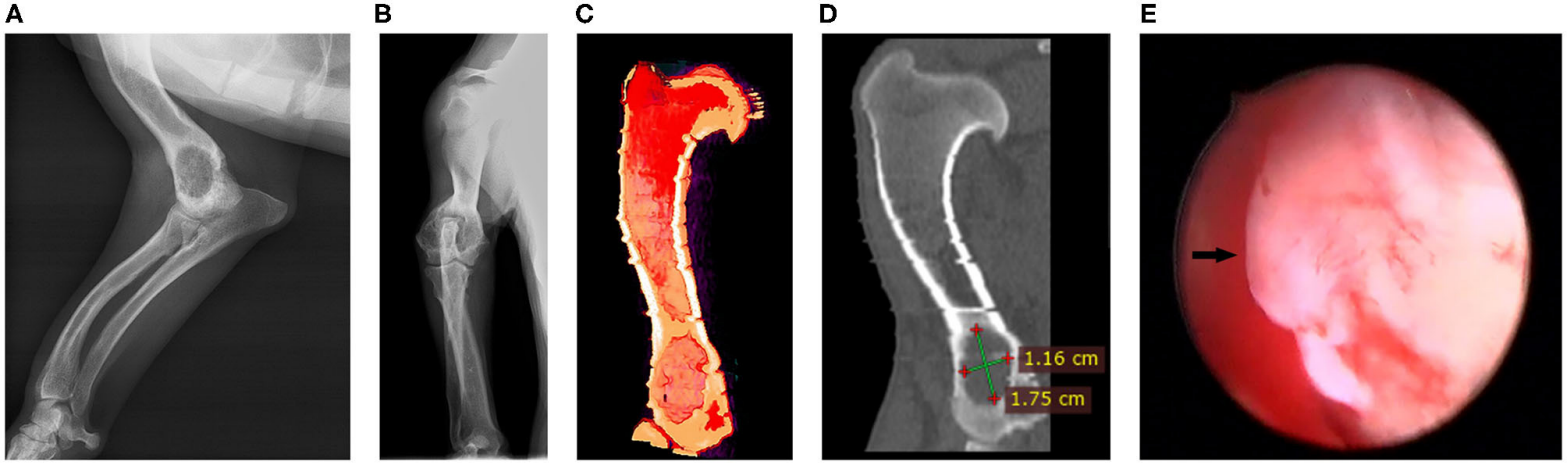
Diagnostic tests. **(A)** Medio-lateral presurgical radiograph. **(B)** Antero-posterior presurgical radiograph. **(C)** Humerus computed tomography 3D view. **(D)** Sagittal section of the humerus computed tomography. **(E)** Arthroscopy, arrow points to the cyst.

Blood analysis and urinalysis did not show any alterations. Radiographs showed a radiolucent area at the level of the lateral condyle of the right humerus that was confirmed by computed tomography (11.6 × 17.5 × 11 mm) (Figure 1), without signs of periosteal reaction. Thoracic radiographs revealed no evidence of metastasis.

**Figure 2:**
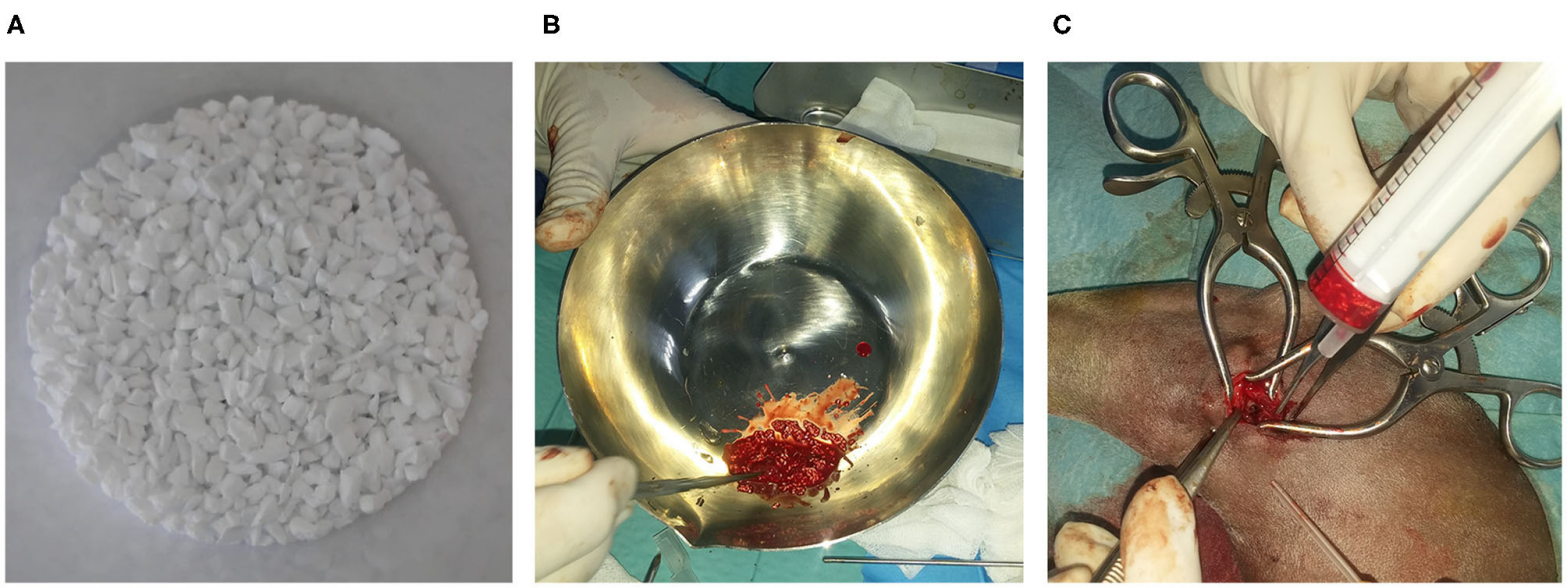
Biomaterial preparation. **(A)** Biomaterial granules. **(B)** Biomaterial mixture with blood of the patient. **(C)** Grafting time.

Suspecting a benign origin, we performed an examination for histopathological evaluation. Using a minimal approach, we introduced an arthroscope to visualize the aspect of the lesion under irrigation (Figure 1 and Supplementary Video 2). Because of a suspected cystic lesion an excisional biopsy was performed, removing the entire lesion and posterior curettage. The defect produced was filled using a marine scaffold (Biofast-Vet) mixed with blood from the patient. Grains of biomaterial between 500 and 1,000 μm in diameter were used (Figure 2).

The extracted material was processed and evaluated by a veterinarian pathologist. Histopathological findings revealed that the cystic wall extracted was composed by a dense connective tissue with low cellularity, and some areas of hyalinized collagen and acellular necrosis. Based on these results, the diagnosis of unicameral bone cyst was confirmed.

**Figure 3:**
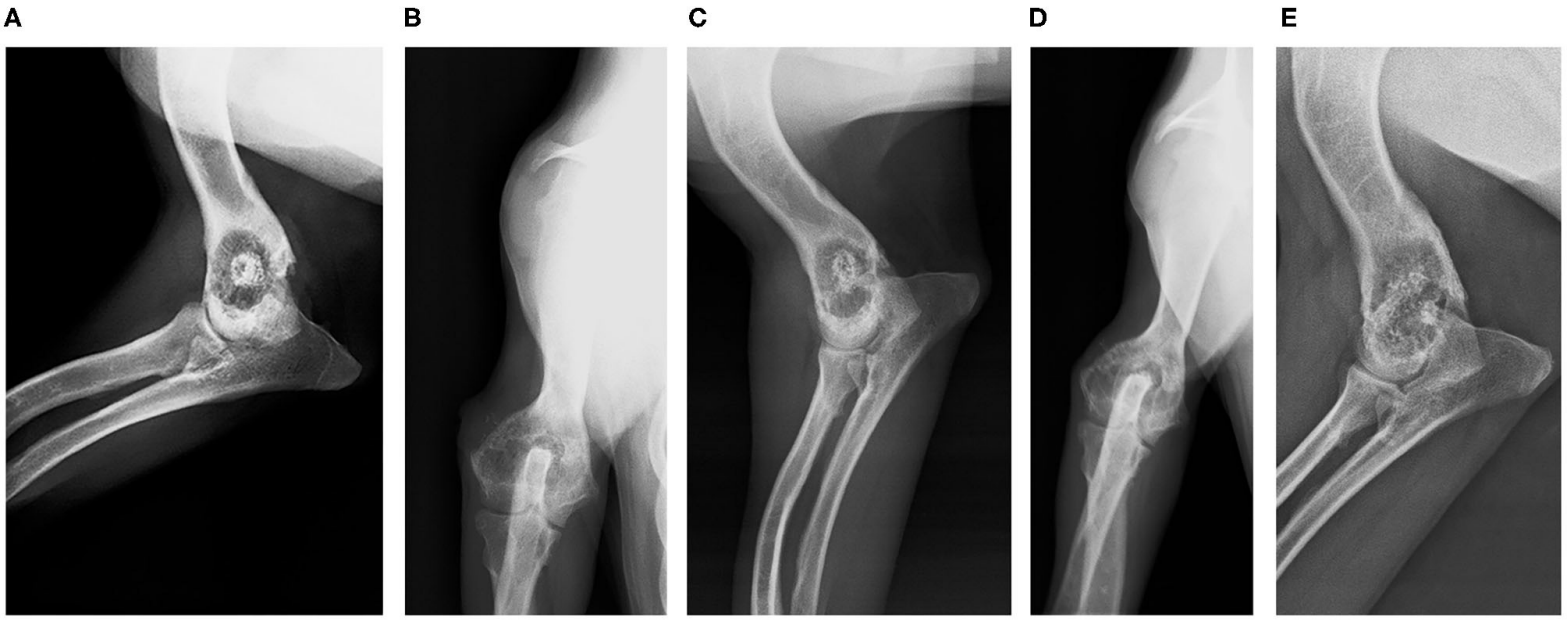
Follow-up radiographs. **(A)** Four-week control (medio-lateral view). **(B)** Four-week control (antero-posterior view). **(C)** Eight-week control (medio-lateral view). **(D)** Eight-week control (antero-posterior view). **(E)** Eighteen-month control (medio-lateral view).

**Figure 4:**
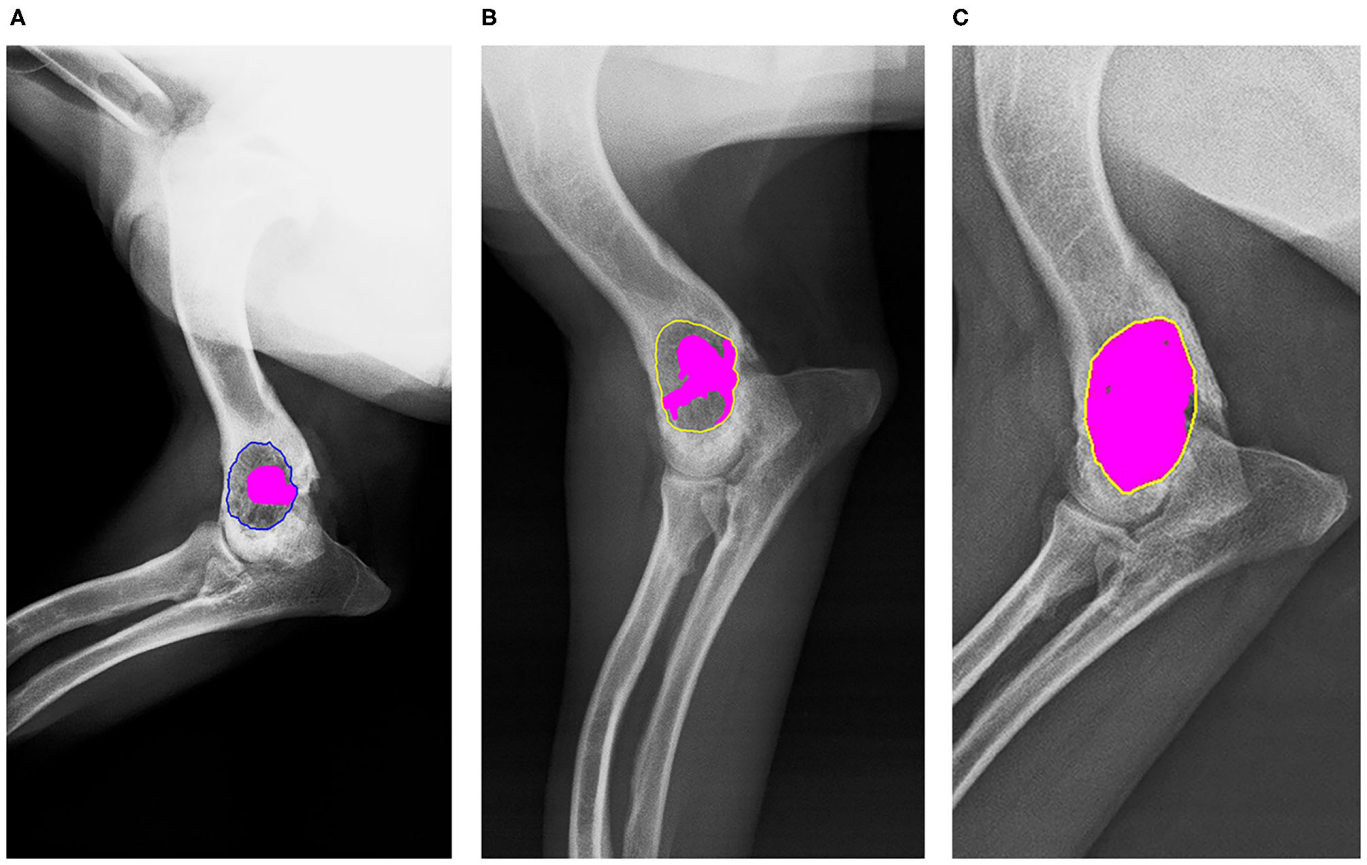
Morphometric radiographic measures. In both, the region of interest (ROI) is the defect, and bone remodeling is painted pink. **(A)** Four-week radiograph (ROI in blue). **(B)** Eight-week radiograph (ROI in yellow). **(C)** Eighteen-month radiograph (ROI in yellow).

Clinical and radiological follow-up was done at 4 weeks, 8 weeks, and 18 months (Figure 3). Each radiograph was evaluated by a stage score from 1 to 5 points (1, not visible callus formation; 2, barely visible callus formation; 3, scattered, not homogeneous calluses; 4, uniform, mature callus formation; 5, very active, hypertrophic callus formation) (17). Bone formation was also quantified morphometrically with Adobe Photoshop CS6 (Adobe, San Jose, CA, USA) and OLYMPUS CellSens Dimension 1.15 (Olympus Corporation, Japan) (Figure 4).

Functional recovery was evaluated using a simple scale (1–15) with three levels: good (11–15), regular (6–10), and poor (1–5), indicating total, partial and no recovery of function, respectively. The criteria that were evaluated were lameness, pain on palpation, and weight bearing (Supplementary Table 1).

Radiographically, the defect was 33.43% remodeled at 4 weeks, 50.24% at 8 weeks, and 94.23 at 18 months (Figure 4). No local or systemic adverse reactions related to the biomaterial have been detected. The functional recovery was good from a few days after the surgery and in the marked follow-up periods it was better. The main clinical sign, lameness, disappeared from the day after surgery.

## Discussion

Bone cysts are usually benign lesions, but sometimes they become malignant or induced secondary fractures (7, 10, 18). Some authors have hypothesized an origin caused by problems of lymphatic drain in the cyst area (19), blood pressure increased (20, 21), or venous stasis (22). Recently, cases in horses have been described that were caused by a deficient blood supply or ischemic cartilage necrosis (21).

The specific pathophysiology of bone cysts is unknown. It is theorized that they can occur as a consequence of trauma, bone hyperplasia, hematoma, or obstruction of blood vessels, triggering an accumulation of fluid in the bone (8). An increment of enzymatic lysosomal activity in the liquid of the cyst may play a role in bone lysis, together with high levels of cytotoxic oxygen free radicals (23).

Clinical signs are usually secondary to progressive expansion of the cyst and includes swelling, pain, lameness especially after a prolonged exercise, and in advanced cases pathologic fractures if the cortical bone becomes very thin (1, 21, 24). In many cases bone cysts are asymptomatic and incidental findings. If clinical signs appear, diagnosis will be focused on imaging methods such as radiography, tomography or magnetic resonance (25–27). Imaging signs show a radiolucent lesion and a cortical thinning. Sometimes a cyst is divided into several compartments separated by trabecular septa (1). Histology of specimens reveals an inner wall composed of a connective tissue rich in collagen fibers, granulation tissue, and multinucleated cells (5, 10). Content is similar to plasma or a serous bloody exudate (5).

Differential diagnosis in dogs include subchondral or aneurysmal bone cysts, bacterial of mycotic infection, fibrous dysplasia, subperiosteal haematoma, or neoplasia (4, 5, 18, 21, 28).

Surgical treatment of symptomatic bone cysts is based in open curettage followed by bone grafting (9, 13). In another studies, it has been described the use of deproteinized bone matrix of animal origin (27). Complete resection of a cyst is recommended but sometimes is not possible, such as with cysts that involve the sesamoid bones (4). In cases where the cyst is large, titanium fixation devices can be used to prevent fracture. Sometimes the consequences of using internal fixation is having to perform arthrodesis, making the patient lose quality of life (8, 9). In human studies, percutaneous injection of corticosteroids (methylprednisolone succinate) has been related to an increment in the levels of prostaglandin E2 in the cystic liquid, having a beneficial effect on the therapy (29). Other studies have also studied synthetic calcium phosphates in mandibular and appendicular defects. Promising results were obtained in terms of speed of bone healing, reduction of the morbidity and improving the quality life of the patients (30).

In other studies, autografts have been used to heal bone defects (31). In a recent study carried out in humans (32), the autograft was compared with a synthetic filler for treatment of unicameral bone cysts, and evidence indicated that the use of the synthetic filler decreased the reintervention rate. In that case, given the size of the defect in relation to the size of the animal, it is very difficult to obtain such a quantity of graft given the morbidity of the donor site, the high risk of infection, and the number of reinterventions.

In the present study, a marine origin bioapatite has been used to reduce the fracture risk during the bone healing, avoiding complications derived from allografts and autografts. The graft is produced as a ceramic material obtained from the use of a fish by-product, shark teeth (*Prionace glauca*). It is a very abundant, low-cost material and eco-friendly biomaterial. In addition, it reduces the risk of disease transmission compared with allografts (13, 14). This material has been preclinically evaluated using the standard of implantable devices for their subsequent use in different clinical cases of veterinary orthopedics such as fractures and arthrodesis. Despite its preclinical nature, in the previous study compared to other similar studies, shorter consolidation times were obtained in dogs with arthrodesis and fractures (16). The use of other marine bioapatites for the treatment of bone defects is unknown at this time.

The limitations of the present study are not being able to perform a CT scan just after surgery and in the last revision. In this way, the density of bone mineralization could have been measured. There is no control to compare with.

## Conclusions

Treatment of bone cysts with marine bioapatite has been effective. The functional recovery of the patient has been good. The lameness disappeared the day after surgery. Moreover, no local or systemic adverse reactions related to the biomaterial have been detected.

## Data Availability Statement

The original contributions presented in the study are included in the article/Supplementary Material, further inquiries can be directed to the corresponding author/s.

## Ethics Statement

The animal study was reviewed and approved by Xunta de Galicia. Written informed consent was obtained from the owners for the participation of their animals in this study.

## Author Contributions

PG and JS obtained and studied the biomaterial and their properties (morphology, characterization, and composition). FdF and TP-E carried out the surgery. MG-G, FM, and AG-C recompiled all information about the case, and analyzed the suitability of the treatment and its correct evaluation. ML-P performed the histopathological evaluation. MG-G carried out the manuscript design and drafted it. All authors have read and approved the final manuscript.

## Conflict of Interest

The authors declare that the research was conducted in the absence of any commercial or financial relationships that could be construed as a potential conflict of interest.
