# Supplementary material for: Case Report: First Evidence of a Benign Bone Cyst in an Adult Teckel Dog Treated With Shark Teeth-Derived Bioapatites
Source: Front Vet Sci. 2021 Feb 22;8:626992. doi: 10.3389/fvets.2021.626992 (PMC7937721; doi:10.3389/fvets.2021.626992)
Supplement: Supplementary file 3 [file Table_1.docx]

| ***Supplementary Table 1.*** *Functionality recovery scoring system for assessing patients* (37,38) | | |
| --- | --- | --- |
| **CRITERIA** | **SCORE** | **CLINICAL EVALUATION** |
| **Lameness** | **1**  **2**  **3**  **4**  **5** | Not walk  Severe limp when walking  Moderate limp when walking  Slight limp when walking  No limp. Walk normally |
| **Pain on palpation** | **1**  **2**  **3**  **4**  **5** | Patient cannot be palpated  Severe signs; patient vocalizeas or becomes aggressive  Moderate signs; patient pulls limb away  Mild signs; patient turns head in recognition  None |
| **Weight-bearing** | **1**  **2**  **3**  **4**  **5** | Non-weight-bearing standing and walking  Partial weight-bearing standing; non-weight-bearing walking  Partial weight-bearing standing; non-weight-bearing walking  Normal standing; favors affected limb when walking  Equal on all limbs standing and walking |
